# Adaptation and validation of the Turkish version of The Brief Screener for Substance and Behavioral Addiction (SSBA)

**DOI:** 10.3389/fpsyg.2025.1697168

**Published:** 2026-01-16

**Authors:** Ardil Bayram Şahin, Arya Yigit, Ceren Turkoglu, Gorkem Emre Oz, Salih Cihat Paltun, Muge Bozkurt, Hale Yapici Eser

**Affiliations:** 1 Graduate School of Health Sciences, Koç University, Istanbul, Türkiye; 2 Research Center for Translational Medicine (KUTTAM), Koç University, Istanbul, Türkiye; 3 School of Medicine, Koç University, Istanbul, Türkiye; 4 Department of Psychiatry, Faculty of Medicine, Istanbul University, Istanbul, Türkiye; 5 Erenkoy Mental Health and Neurological Diseases Training and Research Hospital, Istanbul, Türkiye; 6 Department of Psychiatry, School of Medicine, Koç University, Istanbul, Türkiye

**Keywords:** addiction, behavioral addiction, psychometrics, screening, substance addiction, validation

## Abstract

Behavioral addictions are increasingly recognized due to high prevalence and shared biopsychosocial features with substance use disorders. The Brief Screener for Substance and Behavioral Addictions (SSBA) is a tool designed to assess four substance-related and six behavioral addictions within a unified framework. This study aimed to validate the Turkish version of the SSBA in clinical and general groups (*N* = 193). Internal consistency was excellent (*α* = 0.94 total; ≥0.85 per domain). Exploratory factor analysis largely supported its original ten-factor structure. Convergent and divergent validity were supported by expected correlation patterns. The SSBA effectively distinguished individuals based on engagement and problematic use, with higher scores observed in the clinical group for substance and gambling domains. Overall, Turkish SSBA demonstrates strong psychometric properties, supporting its use as a brief, reliable screening instrument for the assessment of substance and behavioral addictions in clinical practice and research settings.

## Introduction

Addiction is defined as an individual’s urge to engage in substance use or behavior despite its harmful consequences (Clay et al., 2008). While traditionally associated with substance use, certain behavioral addictions that do not involve substances have gained clearer definitions in recent years (Perales et al., 2020). This evolving understanding has shaped diagnostic frameworks such as the Diagnostic and Statistical Manual of Mental Disorders-5 (DSM-5) and the International Classification of Diseases-11 (ICD-11) (American Psychiatric Association, 2013; World Health Organization, 2019). Substance use disorders have been consistently included in all major editions of these two primary diagnostic manuals for psychiatric disorders (American Psychiatric Association, 1980). However, among behavioral addictions, gambling was first recognized in the DSM-3 under the diagnosis of Impulse Control Disorders listed as Pathological Gambling. In the latest editions of the DSM and ICD, it has been reclassified as Gambling Disorder, making it the first behavioral addiction included in the diagnostic classification under the subcategory of Non-Substance-Related Disorders within the Substance-Related and Addictive Disorders section (DSM-5) and the Disorders due to addictive behaviors section (ICD-11). Similarly, ICD-11 includes Gaming Disorder under the Disorders due to Addictive Behaviors section, while the DSM-5 lists Internet Gaming Disorder in the section recommending conditions for further research. ICD-11 further expands this scope by including a diagnosis for Compulsive Sexual Behavior Disorder. Regardless of ongoing debates around classification systems, behavioral addictions may present the outcome of transdiagnostic phenomena representing alterations in reward expectations, reward responses, motivation, cognitive control, and behavior with different stimuli (Brand and Potenza, 2023; Robbins et al., 2012; Yucel et al., 2019). A meta-analysis study that includes studies with different substance use disorders, internet gaming and pathological gambling disorder also revealed increased functional connectivity of clusters including caudate, putamen, thalamus, insula, amygdala and parahippocampal gyrus, but also decreased connectivity of right dorsal anterior cingulate and medial frontal cortex, showing shared neurobiological pathways (Tolomeo and Yu, 2022).

Substance use disorders have been studied more extensively in the literature. Still, as behavioral addictions have become increasingly recognized in recent years, their global prevalence rates have highlighted their significance. A systematic review and meta-analysis analyzing data from 94 studies with 237,657 participants across 40 countries reported the following prevalence rates for behavioral addictions: smartphone (30.7%), food (21%), social media (15.1%), internet (10.6%), sex (9.4%), shopping (7.2%), gambling (7.2%), exercise (7%), and gaming (5.3%) (Alimoradi et al., 2022).

A study reported substance use comorbidity rates of 15% for excessive exercising, 21.3% for excessive sexual behavior, 12.3% for compulsive shopping, 16.3% for problematic video gaming, and 11.7% for compulsive eating (Konkolý Thege et al., 2015). Given the complexity of addiction and its comorbidities, tools that can assess both substance and behavioral addictions together are needed. While separate scales exist for these behavioral addictions, there are limited scales available that evaluate substance and non-substance addictions together. The Brief Screener for Substance and Behavioral Addictions (SSBA), originally developed by Schluter et al. (2020), provides this functionality, serving as a practical tool for assessing both substance and behavioral addictions under one framework. SSBA was developed using signs and symptoms of problematic involvement across 4 substance addictions (alcohol, tobacco, cannabis, cocaine) and 6 behavioral addictions (gambling, shopping, gaming, eating, sex, working). Following psychometric analyses, 4 key indicators, “I did it too much,” “Once I started, I could not stop,” “I felt I had to do it in order to function,” and “I continued to do it, even though it caused problems” were selected for their strong consistency, validity, and ability to represent both substance and behavioral addictions effectively for SSBA (Schluter et al., 2020). These correspond to key ‘use disorder’ features: craving, withdrawal, persistent use despite negative consequences, and loss of control. This screening tool can be used in psychiatric research studies to identify patients with both substance and behavioral addictions, particularly in psychiatric studies focusing on the neurobiology of use disorders.

In this study, it was aimed to conduct a validation study of the Turkish version of SSBA. For this aim, the original English version of the SSBA was translated into Turkish, assessed its internal reliability and factor structure for each target behavior, its convergent and divergent validity in relation to existing screening instruments in a sample of the general population and clinical sample, including multiple types of addictions, were analyzed.

## Materials and methods

### Translation of SSBA

Permission from the developer of the scale was received to translate SSBA into Turkish. The translation process involved four steps: two researchers (ABS and AY) independently translated the English version into Turkish; these translations were reviewed by other researchers and a supervising professor (HYE) to create a single Turkish version. This Turkish version was then checked and finalized by two professors (HYE and MB). Subsequently, a native speaker performed a back-translation into English. The back-translation was compared with the original version, and discrepancies were addressed through discussions among the research team to finalize the Turkish version. The Turkish version of the SSBA scale is presented in the Appendix 1.

### Procedure and study sample

A prior power analysis conducted using G-Power (Version 3.1.9.6) to detect small-to-medium correlations (*ρ* = 0.20) at *α* = 0.05 and 1–*β* = 0.80 indicated that the minimum sample size required is 191 participants. Based on the guideline of recruiting five participants per item, and given that the scale consisted of 40 items, the study aimed to recruit a minimum of 200 participants (Gorsuch, 1983). In this study, there are two sample groups: non-clinical center (Koç University) and four clinical centers, namely the Psychiatry Outpatient Unit at Koç University Hospital, the Psychiatry Department at Istanbul University Faculty of Medicine, the Alcohol and Drug Addiction Research, Treatment and Education Center (AMATEM) at Erenköy Mental Health and Neurological Diseases Training and Research Hospital, and a private outpatient psychiatry clinic, using convenience sampling to ensure a broad representation of individuals with different types of substance and behavioral addictions. Participants were aged between 18 and 65 years, had the capacity to provide informed consent, and were able to understand and write in Turkish. Individuals diagnosed with schizophrenia or dementia, those showing signs of alcohol or substance intoxication, those with an unstable medical condition, or untreated illness that could impair cognition were excluded from the study. These exclusion criteria were assessed using participants’ self-reported medical history in the sociodemographic questionnaire, the attention-check items embedded throughout the survey, and the recorded completion time to ensure reliable responding. For the clinical sample, clinicians referring patients to the study additionally evaluated cognitive impairment and unstable medical conditions as part of routine clinical assessment.

For the non-clinical group, an invitation to participate in the study was sent to students and employees via the Koç University’s official announcement system. Participants were given detailed information about the study upon their interest, and their informed consent was obtained via a separate e-mail. They were then sent an email with a personalized link to participate in the survey by an appointment.

For the clinical sample patients interested in the study were given detailed information about the study, and their written informed consent was obtained. Patients who presented with features of alcohol, nicotine, or substance use disorders based on DSM-5 criteria, as well as behavioral addictions (gaming, gambling, shopping, sexual activity, eating or working) were assessed by a psychiatrist. In the clinical sample, tobacco (71.9%) and alcohol (31.5%) were the most common substance addictions, followed by cannabis (21.3%) and cocaine (5.6%). Gambling (21.3%) and overeating (13.5%) were the most prevalent behavioral addictions; shopping (7.9%), gaming (5.6%), overworking (3.4%), and sex-related addiction (2.2%) were less frequent. Overall, 82% (*n* = 73) reported at least one substance addiction: 34.8% (*n* = 31) had one, 46.1% (*n* = 41) had two, and 1.1% (*n* = 1) had three. Multiple substance addictions were common, while 18% (*n* = 16) had none. Behavioral addictions were reported by 41.6%, with 10.1% (*n* = 9) having two and 1.1% (*n* = 1) three. Both substance and behavioral addictions co-occurred in 23.6% (*n* = 21).

Data collection was conducted using the Qualtrics survey system. Participants were asked to complete the socio-demographic data form and the self-report scales listed below. The survey also included multiple attention-check questions (e.g., “What is 10 + 10?” or “Select ‘A’ option”) to ensure data quality and verify participant attention throughout the survey. Participants who answered the attention-check questions incorrectly had their survey automatically terminated and were not included in the study. Upon completion of the study, all participants received feedback for their individual results via email. Participants were assured that their responses would be kept confidential and would be used only for research purposes. The survey took approximately 30–45 min to complete. All participants were given a gift card worth 100 Turkish Liras (app. 3 Euro). Participants were recruited between March 2023 and December 2024. A total of 216 participants were included in the study. Twenty-three participants were excluded from the analyses: 11 due to incorrect responses to attention-check questions, 3 because of duplicate entries, and 2 due to questionable invariant responses. Additionally, 7 participants’ responses were not included in the analysis due to missing responses coded as ‘I do not know’ or ‘prefer not to say’ in the SSBA. Consequently, the analyses were conducted with 193 participants which satisfied the sample size required for the power analysis.

The study received approval from the Koç University Ethics Committee (Number: 2023.004. IRB2.002 Date: 12.01.2023) and was carried out in accordance with the principles outlined in the Helsinki Declaration.

### Self-report assessments

#### Sociodemographic data form

The sociodemographic data form includes participants’ age, education level, gender, marital status, employment status, monthly income, and presence or family history of psychiatric disorder and physical disease.

#### The Brief Screener for the Substance and Behavioral Addictions

The SSBA is designed to measure the risk of addiction to various substances and behaviors (Schluter et al., 2018, 2020; Hodgins et al., 2023). The SSBA consists of four self-report questions, each reflecting a symptom of problematic engagement (“I did it too much,” “Once I started, I could not stop,” “I felt I had to do it in order to function,” and “I continued to do it, even though it caused problems”). These items are evaluated for four substances (alcohol, nicotine, cannabis, and cocaine) and six behaviors (gambling, shopping, video gaming, eating, sexual activity, and working).

Each item is rated based on its frequency over the past 12 months on a 5-point Likert scale: 0—None of the time, 1—A little of the time, 2—Some of the time, 3—Most of the time, and 4—All of the time. Two additional response options are available: “I did not do this at all” and “Do not know/prefer not to say.” For each substance or behavior, four items are scored (0–4), resulting in a total score ranging from 0 to 16. In the validation study by Hodgins et al. (2023), the four-item SSBA subscales demonstrated good internal consistency, with Cronbach’s alpha values ranging from 0.77 to 0.95.

In this study, the Turkish version of SSBA, as described in translation steps, was used to assess the validity and reliability of the scale.

#### Barratt Impulsivity Scale-11

The Barratt Impulsiveness Scale-11 (BIS-11), developed by Patton et al. (1995) is a 30-item self-report scale designed to assess impulsivity. It consists of three subscales: inattention (8 items), motor impulsivity (11 items), and non-planning impulsivity (11 items). Items are rated on a 4-point Likert scale, with higher scores indicating greater impulsivity. Some items are reverse coded to minimize response bias. The original study reported a Cronbach’s alpha of 0.83, while the Turkish validation study reported a value of 0.78 (Gulec et al., 2008).

#### Convergent validity scales

In this study, a series of measurement tools with established validity and reliability in the literature were used to determine individuals’ risk levels for various types of addiction. The Alcohol Use Disorders Identification Test (AUDIT) (Babor et al., 2001; Saatcioglu et al., 2002) was used to identify problems related to alcohol use, the Fagerström Test for Nicotine Dependence (FTND) (Heatherton et al., 1991; Uysal et al., 2004) was used to assess the physical dimension of nicotine addiction, the Cannabis Use Problems Identification Test (CUPIT) (Bashford et al., 2010; Evren et al., 2017) was used to screen for problematic cannabis use, and the Drug Use Disorders Identification Test (DUDIT) (Berman et al., 2005; Evren et al., 2014) was used to measure problems related to substance use. Additionally, the Problem Gambling Severity Index (PGSI) (Ferris and Wynne, 2001; Arcan, 2020) was used to determine the risk level of gambling behavior, the Compulsive Buying Scale (CBS) (Valence et al., 1988; Yuncu and Kesebir, 2014) was used to assess impulsive buying tendencies, the Game Addiction Scale (GAS) (Lemmens et al., 2009; Akin et al., 2016) was used to measure the risk of computer and video game addiction, and the Modified Yale Food Addiction Scale 2.0 (mYFAS 2.0) (Schulte and Gearhardt, 2017; Tok et al., 2023) to identify symptoms of food addiction, the Sexual Compulsivity Scale (SCS) (Ballester-Arnal et al., 2013; Akin and Celik, 2015) to measure the intensity and lack of control over sexual thoughts and behaviors, and the Bergen Work Addiction Scale (BWAS) (Andreassen et al., 2012; Ozsoy, 2020) to assess tendencies toward work addiction. Each of these instruments has demonstrated strong psychometric properties in previous research, and detailed information regarding their structure, scoring, and reliability is provided in Table 1.

**Table 1:** Features of criterion measurements and Cronbach’s alpha values of all addiction domains in this sample (*n* = 193).

SSBA domains and (*α*)	Scales and (*α*)	Time frame	Item number	Range	Scoring	Cut-off scores	*α* Original/Turkish	References
*Alcohol* (0.88)	AUDIT (*α* = 0.93)	Last 12 months	10	0–40	3–4-point Likert	1–7 = low risk; 8–14 = harmful drinking; ≥13/15 (women/men) = dependence	0.80–0.95/0.81	Babor et al. (2001), and Saatcioglu et al. (2002)
*Tobacco* (0.89)	FTND (*α* = 0.76)	Current status	6	0–10	2–4-point Likert	0–2 = low; 3–4 = low-moderate; 5–7 = moderate; 8–10 = high risk	0.61/0.56	Heatherton et al. (1991), and Uysal et al. (2004)
*Cannabis* (0.95)	CUPIT (*α* = 0.93)	Last 12 months	16	0–82	5–6–7–8–9-point Likert	≥12 problematic use; ≥20 = dependence	0.91/0.89	Bashford et al. (2010), and Evren et al. (2017)
*Cocaine* (0.94)	DUDIT (*α* = 0.94)	Last 12 months	11	0–44	3–4-point Likert	1–5 = low risk; ≥2(women) ≥ 6(men) problematic use; ≥ 25 = dependence	0.93/0.90	Berman et al. (2005), and Evren et al. (2014)
*Gambling* (0.95)	PGSI (*α* = 0.97)	Last 12 months	9	0–27	4-point Likert	1–2 = low risk; 3–7 = moderate risk; ≥8 = problematic level	0.84/0.84	Ferris and Wynne (2001), and Arcan (2020)
*Shopping* (0.85)	CBS (*α* = 0.90)	Current status	12	12–60	5-point Likert	≥42 = addiction	0.0.80/0.85	Valence et al. (1988) and Yuncu and Kesebir (2014)
*Gaming* (0.90)	GAS (*α* = 0.93)	Last 6 months	7	7–35	5-point Likert	≥3 at least 4 items = problematic use; ≥4 at least 4 items = addiction	0.82/0.91	Lemmens et al. (2009), and Akin et al. (2016)
*Overeating* (0.89)	mYFAS 2.0 (0.87)	Last 12 months	13	0–11	8-point Likert	2–3 = mild; 4–5 = moderate; ≥6 = severe	0.86/0.80	Schulte and Gearhardt (2017), and Tok et al. (2023)
*Sexuality* (0.88)	CSS (*α* = 0.88)	Current status	10	10–40	1–4-point Likert	≥24 = addiction	0.84/89	Ballester-Arnal et al. (2013), and Akin and Celik (2015)
*Working* (0.87)	BWAS (*α* = 0.86)	Last 12 months	7	7–35	5-point Likert	≥3 at least 4 items = problematic level; ≥4 at least 4 items = addiction	0.80–0.84/0.86	Andreassen et al. (2012), and Ozsoy (2020)

SSBA, The Brief Screener for Substance and Behavioral Addictions; AUDIT, Alcohol Use Disorders Identification Test; FTND, Fagerstrom Test for Nicotine Dependence CUPIT, Cannabis Use Problems Identification Test; DUDIT, Drug Use Disorders Identification Test; PGIS, Problem Gambling Severity Index; CBS, Compulsive Buying Scale; GAS, Game Addiction Scale; mYFAS 2.0, modified-Yale Food Addiction Scale version 2.0; SCS, Sexual Compulsivity Scale; BWAS, Bergen Work Addiction Scale.

*α*: Cronbach’s alpha.

#### Statistical analysis

Statistical analyses were conducted using IBM SPSS Statistics, version 28. Descriptive statistics were calculated for variables such as age, sex, education level, marital status, employment status, and self-reported psychometric scales. (Table 2).

**Table 2:** Socio-demographic and clinical characteristics of the sample (*n* = 193).

Socio-demographic characteristics	Min–Max	Mean (SD) or *n* (%)
Age	18–59	27.9 (9.3)
Gender		
Woman		97 (% 50.3)
Man		94 (% 48.7)
Non-binary		2 (% 1)
Marital status		
Married		34 (% 17.6)
Non-married/divorced		159 (%82.4)
Employment		
Active worker		70 (% 36.3)
Student		101 (52.3)
Retired		2 (% 1)
Unemployed		20 (10.4)
Education		
Primary/middle school		16 (% 8.4)
High school		89 (% 46.1)
Undergraduate		58 (% 30)
Graduate		30 (% 15.5)
Income (monthly)		
None		37 (% 19.2)
Below minimum wage^a^		67 (% 34.7)
Between 1 and 2 minimum wages		41 (% 21.2)
Above 2 minimum wages		48 (% 24.9)

Measurements	Min–Max	Mean (SD)
Alcohol Use Disorders Identification Test (AUDIT)	1–39	9.47 (9.42)
Fagerstrom Nicotine Dependence Test (FTND)	0–10	4.41 (3.06)
Cannabis Use Problems Identification Test (CUPIT)	3–66	20.91 (16.24)
Drug Use Disorders Identification Test (DUDIT)	0–42	3.80 (8.31)
Problem Gambling Severity Index (PGSI)	0–24	2.35 (5.73)
Compulsive Buying Scale (CBS)	12–56	24.79 (9.55)
Game Addiction Scale (GAS)	7–32	10.38 (5.74)
Modified-Yale Food Addiction Scale 2.0 (mYFAS 2.0)	0–12	1.28 (2.41)
Sexual Compulsivity Scale (SCS)	10–37	15.61 (5.86)
Bergen Work Addiction Scale (BWAS)	7–32	18.03 (6.80)
Barratt Impulsivity Scale-11 (BIS-11)	39–106	62.96 (12.45)

Mean (SD) values of AUDIT, FTND, and CUPIT scores were calculated only among current users who completed these additional related measures (*n* = 156, *n* = 108, and *n* = 56, respectively).

a The average minimum wage was decided at 10.000 Turkish lira.

The Shapiro–Wilk test was used to assess the normality of data distributions. Since the data did not show a normal distribution, non-parametric tests were applied. Continuous variables were compared using the Mann–Whitney U test, while categorical variables were analyzed using either the Chi-square test or Fisher’s exact test.

Spearman’s correlation analysis was employed to examine relationships between numerical variables. The reliability of the SSBA was assessed using Cronbach’s alpha coefficient [with 0.70 considered the acceptable threshold for internal consistency according to Nunnally and Bernstein (1994)], item-total score correlations, and the “Cronbach’s alpha if item deleted” statistic. Item-total correlations of ≥0.30 were considered acceptable, with a minimum threshold of 0.25.

Sampling adequacy was evaluated using the Kaiser–Meyer–Olkin (KMO) measure and Bartlett’s test of sphericity. Confirmatory factor analysis (CFA) based on the original factor structure of the SSBA was conducted using Jamovi software (version 2.5). This was followed by an Exploratory Factor Analysis (EFA) with varimax rotation (with an eigenvalue greater than 1), focusing on items with factor loadings above 0.35.

To assess convergent and divergent validity, Spearman’s correlations were computed between the SSBA and other measures such as AUDIT, FTND, CUPIT, DUDIT, GAS, PGSI, CBS, mYFAS v. 2.0, SCS, BWAS, and BIS-11. A *p*-value strictly less than 0.05 was considered statistically significant.

## Results

### Descriptive characteristics

The final analytic sample included 193 participants. The non-clinical group composed of 108 participants (students/staff), while the clinical group included 85 participants diagnosed with substance use disorders, and/or behavioral addiction features. Of the 193 participants included in the analysis, 50% (*n* = 97) were women, 49% (*n* = 94) were men, and 1% (*n* = 2) identified as non-binary. The mean age of the participants was 27.9 (SD = 9.3) years, with an age range of 18–59 years (Table 2).

### Clinical characteristics of the sample

Participants’ addiction profiles showed wide variability: 19.2% reported no alcohol use (AUDIT = 0) while 15% met criteria for alcohol use disorder (AUDIT ≥ 15); 44% did not use tobacco (FTND = 0) whereas 11.4% were high-risk smokers (FTND = 8–10); 71% did not use cannabis (CUPIT = 0) while 13% were addicted (CUPIT ≥ 20); 69.9% did not use stimulants (score = 0) while 4.7% were addicted (score ≥ 25); 11.9% showed problematic gambling (PGSI ≥ 8); 7.3% met criteria for compulsive buying addiction (CBS ≥ 42); 5.2% were addicted to gaming (GAS ≥ 4 with ≥3 items); 6.7% had severe addictive eating (mYFAS v. 2.0 ≥ 6); 13.5% met criteria for compulsive sexual behavior addiction (SCS ≥ 24); and 25.9% were classified as addicted to overworking (BWAS ≥ 4 with ≥3 items).

Table 2 presents a detailed overview of the clinical characteristics and socio-demographic information of the sample.

### Internal consistency and reliability

The SSBA demonstrated excellent internal consistency with a total Cronbach’s alpha of 0.94, with values ranging from 0.93 to 0.94. Item-total correlations were mostly greater than 0.3, except for the first two questions for overworking. Cronbach’s alpha value ranged from 0.85 to 0.95 in the analyses conducted for each domain. The SSBA total, all domains and items Cronbach’s alpha values exceeding 0.70, the scale demonstrated a strong reliability. Table 3 presents the internal consistency results, and Table 1 provides Cronbach’s alpha values for each of the ten SSBA domains.

**Table 3:** SSBA four items, ten target behaviors, internal consistency, and Cronbach’s alpha values of all items (*n* = 193).

	Item-total correlation	Cronbach’s alpha if item deleted
1. Think about the statement, “I did it too much.” In the last 12 months, how often did this apply?		
Alcohol	0.28	0.94
Tobacco	0.39	0.94
Cannabis	0.58	0.94
Cocaine	0.66	0.94
Gambling	0.65	0.94
Shopping	0.28	0.94
Gaming	0.41	0.93
Overeating	0.31	0.94
Sexuality	0.35	0.94
Overworking	0.16	0.94
2. Think about the statement, “Once I started, I could not stop.” In the last 12 months, how often did this apply?		
Alcohol	0.34	0.94
Tobacco	0.54	0.94
Cannabis	0.68	0.94
Cocaine	0.74	0.93
Gambling	0.74	0.93
Shopping	0.5	0.94
Gaming	0.54	0.94
Overeating	0.36	0.94
Sexuality	0.44	0.94
Overworking	0.21	0.94
3. Think about the statement, “I felt I had to do it in order to function.” In the last 12 months, how often did this apply?		
Alcohol	0.35	0.94
Tobacco	0.51	0.94
Cannabis	0.66	0.94
Cocaine	0.75	0.93
Gambling	0.73	0.94
Shopping	0.55	0.94
Gaming	0.6	0.94
Overeating	0.44	0.94
Sexuality	0.45	0.94
Overworking	0.31	0.94
4. Think about the statement, “I continued to do it, even though it caused problems.” In the last 12 months, how often did this apply?		
Alcohol	0.38	0.94
Tobacco	0.56	0.94
Cannabis	0.64	0.94
Cocaine	0.74	0.93
Gambling	0.7	0.94
Shopping	0.52	0.94
Gaming	0.58	0.94
Overeating	0.37	0.94
Sexuality	0.46	0.94
Overworking	0.34	0.94
Total SSBA		0.94

SSBA, The Brief Screener for Substance and Behavioral Addictions.

### Factor analysis

Confirmatory Factor Analysis (CFA) based on the item distributions of the original scale was first performed to test the validity of the factor structure of the measurement tool used in our study. However, the model fit indices obtained showed that the theoretical structure proposed for our current data set could not be sufficiently validated. Specifically, values such as CFI = 0.833, TLI = 0.813, RMSEA = 0.0965 (90% CI: 0.0915–0.102), and *χ*^2^/df ≈ 2.8 indicate that the model fit is weak. Therefore, EFA was applied to redefine the factor structure in a manner appropriate for our dataset. The KMO measure of sampling adequacy was excellent at 0.85, and Bartlett’s test of sphericity demonstrated strong statistical significance [RMSEA = 0.137 (90% CI: 0.132–0.143), TLI = 0.576, *χ*^2^ = 2,569, df = 555, *p* < 0.001], indicating the suitability of the data for factor analysis. Minimum residual extraction method with Varimax rotation revealed a ten-factor solution that accounted for 74.8% of the total variance and clearly reflected the multidimensional structure of the SSBA. Factor loadings indicated distinct addiction domains: cannabis and cocaine (Factor 1), gaming (Factor 2), alcohol (Factor 3), eating (Factor 4), gambling (Factor 5), sexuality (Factor 6), working (Factor 7), shopping (Factor 8), and tobacco (Factor 9). Factor 10 lacked clear interpretability due to inconsistent and relatively low item loadings (Supplement 1).

### Discriminative validity of SSBA for differentiating according to engagement and problematic patterns

First, participants exhibiting addictive behaviors were compared with those not exhibiting such behaviors across all SSBA domains. Participation was defined as scoring at least 1 point on the relevant scales, indicating engagement in the corresponding addictive behavior. The behaviors with the highest average engagement were tobacco use (Mean = 6.02, SD = 5.78), eating (Mean = 5.11, SD = 4.23), and working (Mean = 4.76, SD = 4.39). In contrast, the addictive behaviors with the lowest average engagement were cocaine use (Mean = 0.37, SD = 1.78) and cannabis use (Mean = 1.45, SD = 3.23). Mann–Whitney U tests revealed significant differences in SSBA scores between participants who engaged in addictive behaviors and those who did not, indicating the scale’s sensitivity to behavioral engagement (*p* < 0.001 across all addictive behaviors except *p* = 0.036 for the shopping domain) (Table 4).

**Table 4:** Descriptive features of SSBA all target behaviors, comparison of all target behaviors of the SSBA between participants engaged and non-engaged in these behaviors in the past 12 months.

SSBA domains	All sample			Cut-off scores of related scales	Behavior engagement in past 12 months^a^			No behavior engagement in past 12 months			*Z*	*p*
	*N*	Range	Mean (SD)		*N*	Range	Mean (SD)	*N*	Range	Mean (SD)		
Alcohol	193	0–16	4.19 (4.33)	AUDIT≥1	156	0–16	5.07 (4.31)	37	0–9	0.46 (1.56)	−8.199	**<0.001**
Tobacco	193	0–16	6.02 (5.78)	FGS ≥ 1	108	0–16	10.15 (4.27)	85	0–10	0.76 (1.77)	−11.509	**<0.001**
Cannabis	193	0–15	1.45 (3.23)	CUPIT≥1	56	0–15	4.86 (4.40)	137	0–4	0.06 (0.38)	−12.318	**<0.001**
Cocaine	193	0–15	0.37 (1.78)	DUDIT≥1	58	0–16	1.21 (3.11)	135	0–1	0.01 (0.09)	−5.811	**<0.001**
Gambling	193	0–16	1.69 (3.82)	PGSI≥1	44	0–16	7.07 (5.12)	149	0–4	0.10 (0.42)	−11.732	**<0.001**
Shopping	193	0–16	4.49 (3.49)	CBS ≥ 13	187	0–16	4.57 (3.50)	6	3-Jan	1.83 (0.98)	−2.098	**0.036**
Gaming	193	0–16	2.82 (3.79)	GAS≥8	86	0–16	5.50 (4.22)	107	0–6	0.67 (1.13)	−9.397	**<0.001**
Eating	193	0–16	5.11 (4.23)	mYFAS 2.0 ≥ 1	64	0–16	8.92 (3.72)	129	0–12	3.22 (3.04)	−8.463	**<0.001**
Sexuality	193	0–16	3.35 (3.52)	SCS ≥ 11	158	0–16	3.88 (3.56)	35	0–8	0.94 (2.11)	−5.396	**<0.001**
Working	193	0–16	4.76 (4.39)	BWAS≥8	181	0–16	5.02 (4.41)	12	0–4	0.92 (1.24)	−3.433	**<0.001**

SSBA, The Brief Screener for Substance and Behavioral Addictions; AUDIT, Alcohol Use Disorders Identification Test; FTND, Fagerstrom Test for Nicotine Dependence; CUPIT, Cannabis Use Problems Identification Test; DUDIT, Drug Use Disorders Identification Test; PGIS, Problem Gambling Severity Index; CBS, Compulsive Buying Scale; GAS, Game Addiction Scale; mYFAS 2.0, modified-Yale Food Addiction Scale version 2.0; SCS, Sexual Compulsivity Scale; BWAS, Bergen Work Addiction Scale; SD, Standard deviation; Mann Whitney U Test. Significant values marked bold *p* < 0.05.

a Behavior engagement in past 12 months descriptive statistics include participants who had indicated they had engaged in the target behavior in the past 12 months and completed additional criterion scale for those addictions.

Second, participants with and without problematic addictive behaviors were compared across all SSBA domains. Problematic patterns of addictive behavior were identified using predefined cut-off values derived from related scales. Mann–Whitney U tests demonstrated statistically significant differences in SSBA scores between participants with and without problematic addictive behaviors (*p* < 0.001 across all behaviors) (Table 5).

**Table 5:** Comparison of all target behaviors of the SSBA between participants with and without problematic addictive features in the past 12 months (*n* = 193).

SSBA domains	Cut-off scores of related scales	Problematic addictive features in past 12 months^a^			No problematic addictive features in past 12 months			*Z*	*p*
		*N*	Range	Mean (SD)	*N*	Range	Mean (SD)		
Alcohol	AUDIT≥8	66	1–16	8.39 (4.42)	127	0–10	2.00 (2.09)	−9.33	**<0.001**
Tobacco	FGS ≥ 3	74	2–16	11.62 (3.53)	119	0–16	2.53 (3.82)	−10.408	**<0.001**
Cannabis	CUPIT≥12	33	1–15	7.42 (4.01)	160	0–4	0.22 (0.63)	−11.042	**<0.001**
Cocaine	DUDIT≥6	36	0–15	1.92 (3.78)	157	0–1	0.01 (0.11)	−7.424	**<0.001**
Gambling	PGSI≥8	23	2–16	10.96 (3.77)	170	0–7	0.44 (1.21)	−9.777	**<0.001**
Shopping	CBS ≥ 42	14	2–16	10.36 (4.14)	179	0–13	4.03 (2.99)	−4.733	**<0.001**
Gaming	GAS≥3 in ≥4 items	25	1–16	8.56 (4.21)	168	0–12	1.97 (2.88)	−6.260	**<0.001**
Eating	mYFAS 2.0 ≥ 2	45	0–16	9.62 (3.78)	148	0–12	3.74 (3.32)	−7.505	**<0.001**
Sexuality	SCS ≥ 24	26	1–16	6.54 (3.67)	167	0–14	2.85 (3.24)	−4.885	**<0.001**
Working	BWAS≥3 in ≥4 items	101	0–16	6.78 (4.26)	92	0–15	2.54 (3.36)	−6.961	**<0.001**

SSBA, The Brief Screener for Substance and Behavioral Addictions; AUDIT, Alcohol Use Disorders Identification Test; FTND, Fagerstrom Test for Nicotine Dependence; CUPIT, Cannabis Use Problems Identification Test; DUDIT: Drug Use Disorders Identification Test, PGIS: Problem Gambling Severity Index, CBS: Compulsive Buying Scale, GAS: Game Addiction Scale, mYFAS 2.0: modified-Yale Food Addiction Scale version 2.0, SCS: Sexual Compulsivity Scale; BWAS, Bergen Work Addiction Scale; SD, Standard deviation; Mann Whitney U Test. Significant values marked bold *p* < 0.05.

a Problematic addictive features in past 12 months include participants who had indicated they had engaged in the target behavior in problematic level based on cut-off value based on related criterion measurements.

Mann–Whitney U tests confirmed the SSBA’s ability to discriminate between participants with and without engagement and problematic engagement in target behaviors. These analyses of participation in addictive behaviors were assessed with SSBA to determine the extent and severity of substance use and behavioral addictions within the sample.

Third and finally, the general and clinical samples across all SSBA domains were compared. Comparison of clinical and general samples revealed significant differences in SSBA scores, with clinical participants displaying substantially higher scores in alcohol, tobacco, cannabis, cocaine, and gambling use (*p* < 0.001). The clinical group also showed higher, scores for sexual addictive behaviors (Mean = 4.02 vs. 2.81; *p* = 0.047). In contrast, no significant differences were observed between clinical and general samples for shopping, gaming, eating, and working behaviors, despite slightly higher scores in the general population for shopping, eating, and working domains (all *p* > 0.05). (Table 6).

**Table 6:** Comparison of all target behaviors of the SSBA between clinical sample and general sample (*n* = 193).

SSBA	General sample			Clinical sample			*Z*	*p*
	*N*	Range	Mean (SD)	*N*	Range	Mean (SD)		
Alcohol	108	0–13	2.90 (2.80)	85	0–16	5.82 (5.29)	−3.501	**<0.001**
Tobacco	108	0–16	3.70 (4.85)	85	0–16	8.95 (5.54)	−5.972	**<0.001**
Cannabis	108	0–5	0.33 (0.95)	85	0–15	2.87 (4.36)	−4.949	**<0.001**
Cocaine	108	0–2	0.03 (0.21)	85	0–15	0.80 (2.62)	−3.673	**<0.001**
Gambling	108	0–7	0.53 (1.40)	85	0–16	3.16 (5.19)	−3.454	**<0.001**
Shopping	108	0–13	4.62 (3.02)	85	0–16	4.32 (4.01)	−1.55	0.121
Gaming	108	0–12	2.20 (3.00)	85	0–16	3.61 (4.50)	−1.92	0.055
Eating	108	0–16	5.29 (3.87)	85	0–16	4.89 (4.67)	−1.341	0.18
Sexuality	108	0–11	2.81 (3.05)	85	0–16	4.02 (3.96)	−1.985	**0.047**
Working	108	0–15	4.87 (4.22)	85	0–16	4.62 (4.63)	−0.8	0.424

SSBA, The Brief Screener for Substance and Behavioral Addictions; AUDIT, Alcohol Use Disorders Identification Test; FTND, Fagerstrom Test for Nicotine Dependence; CUPIT, Cannabis Use Problems Identification Test; DUDIT, Drug Use Disorders Identification Test; PGIS, Problem Gambling Severity Index; CBS, Compulsive Buying Scale; GAS, Game Addiction Scale; mYFAS 2.0, modified-Yale Food Addiction Scale version 2.0; SCS, Sexual Compulsivity Scale; BWAS, Bergen Work Addiction Scale; SD, Standard Deviation; Mann Whitney U Test. Significant values marked bold *p* < 0.05.

### Convergent and divergent validity analysis of SSBA with criterion measurements

Correlation matrix was used to examine convergent and divergent validity based on a Spearman correlation. The correlations between SSBA and related criterion measures, and the Barratt impulsivity scale for convergent validity, were investigated. SSBA domains showed significant positive correlations with related criterion measures, confirming strong convergent validity. Significant correlations were found between the BIS-11 and all subdomains, ranging from 0.154 (cocaine, *p* < 0.05) to 0.353 (sexual activity, *p* < 0.001), except working (*r* = −0.031, *p* = 0.673). Correlations between all SSBA target domain scores and the criterion measures demonstrated significant relationships, presented in Table 7 as a heatmap.

**Table 7:** Heatmap representation of correlation analysis of SSBA all target behavior and other criterion scales.

SSBA	AUDIT	FTND	CUPIT	DUDIT	PGSI	CBS	GAS	mYFAS2.0	SCS	BWAS	BIS-11	Scale
Alcohol	**0.763**	**0.246**	−0.052	**0.184**	0.044	0.101	−0.041	−0.054	**0.257**	0.007	**0.217**	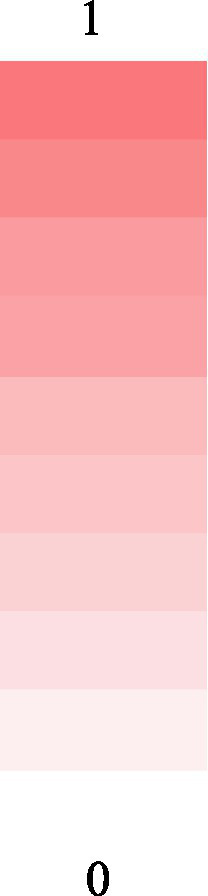
Tobacco	**0.34**	**0.575**	0.245	**0.376**	0.079	0.083	−0.007	−0.071	0.109	0.064	**0.192**	
Cannabis	**0.276**	**0.242**	**0.89**	**0.825**	−0.04	−0.019	0.048	−0.088	0.121	−0.048	**0.163**	
Cocaine	0.145	**0.254**	**0.339**	**0.508**	0.064	**0.159**	0.036	0.049	0.057	−0.118	**0.154**	
Gambling	−0.002	0.182	0.1	−0.018	**0.868**	0.006	**0.278**	−0.06	0.121	0.003	**0.219**	
Shopping	−0.018	0.099	−0.064	−0.101	0.029	**0.531**	−0.016	**0.294**	0.065	**0.196**	**0.172**	
Gaming	0.017	0.122	0.035	0.013	0.252	0.055	**0.741**	−0.007	**0.238**	−0.059	**0.248**	
Eating	−0.15	−0.135	−0.114	−0.072	0.023	**0.387**	**0.15**	**0.657**	0.047	0.061	**0.281**	
Sexuality	**0.268**	0.177	0.082	**0.239**	0.127	**0.238**	**0.268**	0.077	**0.603**	0.049	**0.353**	
Working	0.08	0.107	0.178	0.065	0.028	**0.151**	−0.073	0.019	0.084	**0.557**	−0.031	

SSBA, The Brief Screener for Substance and Behavioral Addictions; AUDIT, Alcohol Use Disorders Identification Test; FTND, Fagerstrom Test for Nicotine Dependence; CUPIT, Cannabis Use Problems Identification Test; DUDIT, Drug Use Disorders Identification Test; PGIS, Problem Gambling Severity Index; CBS, Compulsive Buying Scale; GAS, Game Addiction Scale; mYFAS 2.0, modified-Yale Food Addiction Scale version 2.0; SCS, Sexual Compulsivity Scale; BWAS, Bergen Work Addiction Scale; BIS11, Barratt Impulsivity Scale; M, Mean; SD, Standard Deviation. *n* = 193, Mean and SD values of AUDIT, FTND and CUPIT scores were calculated only among relevant users (*n* = 156, *n* = 108, *n* = 56, respectively). Significant values marked bold *p* < 0.05.

## Discussion

This study aimed to validate the Turkish version of the SSBA and assess its psychometric properties in a diverse sample. Removing individual items had negligible impact on overall reliability, reinforcing that each item significantly contributes to the scale’s construct validity. The SSBA demonstrated excellent psychometric properties, including high internal consistency, strong criterion validity, and effective discriminative validity across various substance and behavioral addiction domains. These results establish the SSBA as a reliable and valid tool for assessing addiction-related behaviors.

The observed addiction profiles in our sample demonstrate considerable heterogeneity across both substance and behavioral domains, partially reflecting broader trends [World Health Organization, 2024; Substance Abuse and Mental Health Services Administration, 2023; Turkish Monitoring Center for Drugs and Drug Addiction (TUBIM), 2019; Gorgulu et al., 2016]. Substance addictions can sometimes co-occur with behavioral addictions or occur independently (Sussman et al., 2011). In the clinical sample, our findings reflect this, with almost a quarter of participants having both substance and behavioral addictions. These findings suggest that co-occurrence of multiple addictive behaviors—particularly multiple substance use—is highly prevalent in this clinical sample, underlining the need for integrated assessment and intervention strategies that consider poly-addiction rather than single-disorder approaches. This pattern emphasizes the importance of screening for comorbid addictions to develop more comprehensive and effective treatment plans for individuals presenting to psychiatric services. Moreover, this pattern may reflect the treatment-seeking population’s primary focus on substance use, whereas behavioral addictions are either underreported or underdiagnosed. Behavioral addictions in our sample showed considerable variability and were partially consistent with the national estimates. In a study conducted among Turkish university students, Akpinar Aslan et al. (2021) reported that the rates of high risk for compulsive shopping, problematic social media use, food addiction, gaming addiction, and sex/pornography addiction were 2.0, 3.6, 3.4, 4.9, and 5.6%, respectively, underlining the importance of screening for various behavioral addictions in this population. In addition, global meta-analyses have reported prevalence rates of 6% for internet addiction (Cheng and Li, 2014), about 4.6% for gaming addiction (Fam, 2018), *0.12–5.8%* for gambling addiction (Calado and Griffiths, 2016), 5% for shopping addiction (Maraz et al., 2016), and approximately 16.2% for food addiction (Burrows et al., 2018). The relatively higher and lower prevalence of some addictions in our sample may reflect the inclusion of clinically diagnosed individuals alongside a university-based population. It is noteworthy that prevalence estimates were based on validated self-report measures and standardized cut-offs, capturing both clinical and non-clinical participants. Among behavioral addictions, work addiction had the highest rate in our sample. This may be partly population-related, as Griffiths et al. (2018) emphasize that work addiction, unlike most other behavioral addictions, can also yield socially acceptable outcomes such as increased productivity, higher income, and social recognition. On the other hand, currently illegal substances such as cannabis and cocaine may have been underreported among non-clinical respondents due to legal concerns. Overall, these descriptive findings reinforce the importance of comprehensive screening tools like the SSBA for accurately capturing and monitoring a broad spectrum of addictive behaviors across diverse populations, aiding in early detection, targeted interventions, and public health planning.

The SSBA demonstrated excellent internal consistency, with Cronbach’s *α* = 0.94 for the total score and ≥0.85 for each domain. This is consistent with Hodgins et al. (2023), who reported high internal reliability of the SSBA among university students, supporting its applicability in young adult populations. All item-total correlations were moderate to high, except for the first two items related to work addiction (“I did it too much” and “Once I started, I could not stop”), which may not adequately capture the compulsive or perfectionistic traits typical of overworking. These findings likely reflect the socially reinforced nature of high work engagement (Griffiths et al., 2018) and suggest that future revisions may benefit from more targeted item development to better capture work addiction.

Factor Analysis results revealed a clear multidimensional structure, consistent with theoretical expectations. Exploratory factor analysis largely supported the original ten-factor structure, with strong evidence for eight distinct factors and a theoretically meaningful merging of cannabis and cocaine items onto a single factor, suggesting overlap in behavioral or neurobiological mechanisms—possibly due to their shared classification as psychoactive substances— and also reflecting the small number of cocaine users in the sample. This finding aligns with Konkolÿ Thege et al. (2023), who demonstrated that the generalized version of the SSBA (SSBA-G) effectively captures a unified addiction construct, supporting a transdiagnostic approach to assessment. The tenth factor was uninterpretable and lacked meaningful item loadings; still, we suggest researchers use the original 10 factor structure for their research while using the Turkish version too.

Discriminative validity analyses confirmed that the SSBA effectively differentiated individuals based on their engagement in and severity of addictive behaviors. Participants with addictive behaviors had significantly higher SSBA scores. The clinical group showed significantly higher scores for substance use and gambling addiction, while gaming addiction was higher in the clinical group, reaching the threshold for clinical significance. Interestingly, the averages for shopping, eating, and working addictions were higher in the general population, but no significant group differences. This pattern highlights that certain behavioral addictions may be equally, or even more prominent in community samples, despite not reaching clinical attention. This may reflect the lower functional impairment and higher social acceptability of behavioral addictions, which can delay clinical recognition. Our findings emphasize that while behavioral addictions may not always prompt treatment-seeking, they are prevalent and clinically relevant. The SSBA’s sensitivity highlights its potential as a valuable screening tool, especially in non-clinical populations. Greater clinical and public health attention should be directed toward behavioral addictions, even when impairment is subtle or help-seeking is absent.

Our study provided robust evidence for convergent validity by demonstrating significant correlations between SSBA subdomains and established addiction-related constructs measured by the criterion scale. Specifically, significant positive correlations were observed between SSBA scores and the Barratt Impulsivity Scale (BIS-11), reinforcing impulsivity as a fundamental risk factor associated with addictive behaviors (McMullin et al., 2021; Lee et al., 2019). Previous literature consistently supports impulsivity’s role in addiction, proposing that higher impulsivity may predispose individuals to engage in risky and addictive behaviors by diminishing inhibitory control and increasing reward sensitivity (Verdejo-Garcia and Albein-Urios, 2021; Mitchell and Potenza, 2014). Notably, working addiction did not significantly correlate with impulsivity, suggesting distinct underlying mechanisms that warrant further exploration, potentially reflecting distinct psychological underpinnings such as compulsivity, perfectionism, or occupational stress rather than impulsivity-driven mechanisms (Kun et al., 2021). The absence of significant correlation between working addiction and impulsivity, as well as relatively low correlations observed in certain addiction domains, may indicate that some addictions are driven more by compulsivity rather than impulsivity. Previous literature supports behavioral addictions, which often involve impulsive reward-seeking behaviors (Grant and Potenza, 2004; Fineberg et al., 2014; Chamberlain and Grant, 2019). Moreover, recent evidence emphasizes that addiction cannot solely be explained by impulsivity; other psychological constructs such as compulsivity, negative reinforcement, and affective dysregulation play crucial roles in maintaining certain addictive behaviors (el-Guebaly et al., 2012; Yucel et al., 2019). Thus, the limited or absent correlations between impulsivity and specific addiction domains, such as working addiction, further highlight the multidimensional complexity of addictive behaviors. However, working addiction may be more related to obsessive compulsive personality traits and may reflect the effect of perfectionism (Kun et al., 2021).

This study had several limitations that should be carefully considered when interpreting the findings. Firstly, the relatively modest sample size may limit the generalizability of our findings to broader populations. The cross-sectional study design precludes causal inference, highlighting the necessity of longitudinal studies to clarify causal relationships between SSBA scores and addiction-related outcomes. Additionally, the reliance on self-reported measures may introduce response bias, as participants might underreport or overreport symptoms due to social desirability or stigma. The absence of structured clinical interviews to confirm diagnostic status might have affected the accuracy of classification between clinical and non-clinical groups. Cultural differences in the expression and perception of addictions were not directly examined, potentially limiting the generalizability to diverse cultural contexts. Lastly, this study did not evaluate test–retest reliability, an essential psychometric property necessary to confirm the stability and consistency of the SSBA across time. Future research should address these limitations through larger, longitudinally designed studies employing clinical diagnostic interviews and diverse cultural sampling. The validated Turkish version of the SSBA offers a reliable, concise screening tool for clinicians and researchers to assess and identify substance and behavioral addictions efficiently. Its cross-cultural reliability further enables international comparisons and contributes significantly to addiction research and practice.

In conclusion, the Turkish version of the SSBA demonstrates excellent psychometric properties, indicating it is a reliable, valid, and efficient instrument for assessing a wide array of substance-related and behavioral addictions. Its strong internal consistency, construct validity, and discriminative capabilities highlight its utility in both clinical practice and addiction research.

## Data Availability

The raw data supporting the conclusions of this article will be made available by the authors, without undue reservation.
