# Supplementary material for: Adaptation and validation of the Turkish version of The Brief Screener for Substance and Behavioral Addiction (SSBA)
Source: Front Psychol. 2026 Jan 16;16:1697168. doi: 10.3389/fpsyg.2025.1697168 (PMC12855089; doi:10.3389/fpsyg.2025.1697168)
Supplement: Supplementary file 1 [file Table_1.docx]

**Supplement 1**

Table S1. Factor Loadings from Exploratory Factor Analysis for ten domains of SSBA in jamovi.

| SSBA domains | Factor | | | | | | | | | |  |
| --- | --- | --- | --- | --- | --- | --- | --- | --- | --- | --- | --- |
|  | 1 | 2 | 3 | 4 | 5 | 6 | 7 | 8 | 9 | 10 |  |
| 1. Alcohol   Item 1  Item 2  Item 3  Item 4 |  |  | **0.704 0.853**  **0.739 0.822** |  |  |  |  |  |  |  |  |
| 1. Tobacco   Item 1  Item 2  Item 3  Item 4 |  |  |  |  |  |  |  |  | **0.800 0.764 0.479**  **0.723** |  |  |
| 1. Cannabis   Item 1  Item 2  Item 3  Item 4 | **0.777**  **0.894**  **0.929**  **0.920** |  |  |  |  |  |  |  |  |  |  |
| 1. Cocaine   Item 1  Item 2  Item 3  Item 4 | **0.689**  **0.788**  **0.806**  **0.765** |  |  |  |  |  |  |  |  |  |  |
| 1. Gambling   Item 1  Item 2  Item 3  Item 4 | 0.414 0.476  0.491 0.473 |  |  |  | **0.698**  **0.759**  **0.715 0.729** |  |  |  |  |  |  |
| 1. Shopping   Item 1  Item 2  Item 3  Item 4 |  |  |  |  |  |  |  | **0.559**  **0.783 0.693 0.683** |  |  |  |
| 1. Gaming   Item 1  Item 2  Item 3  Item 4 |  | **0.699**  **0.835**  **0.721 0.796** |  |  |  |  |  |  |  |  |  |
| 1. Eating   Item 1  Item 2  Item 3  Item 4 |  |  |  | **0.800**  **0.877**  **0.503 0.824** |  |  |  |  |  |  |  |
| 1. Sexuality   Item 1  Item 2  Item 3  Item 4 |  |  |  |  |  | **0.770**  **0.842**  **0.707 0.745** |  |  |  |  |  |
| 1. Working   Item 1  Item 2  Item 3  Item 4 |  |  |  |  |  |  | **0.786**  **0.804 0.766 0.753** |  |  |  |  |

*SSBA:* The Brief Screener for Substance and Behavioral Addictions.

Note. 'Minimum residual' extraction method was used in combination with a varimax rotation.

Table S2. Factor loadings and variance of the items.

| Factor | **SS Loadings** | **% of Variance** | **Cumulative %** |
| --- | --- | --- | --- |
| 1  2  3  4  5  6  7  8  9  10 | 6.948  3.007  2.925  2.856  2.826  2.762  2.657  2.629  2.351  0.971 | 17.37  7.52  7.31  7.14  7.07  6.90  6.64  6.57  5.88  2.43 | 17.4  24.9  32.2  39.3  46.4  53.3  60.0  66.5  72.4  74.8 |

SS: Summary squared.
