# Supplementary material for: Adaptation and validation of the Turkish version of The Brief Screener for Substance and Behavioral Addiction (SSBA)
Source: Front Psychol. 2026 Jan 16;16:1697168. doi: 10.3389/fpsyg.2025.1697168 (PMC12855089; doi:10.3389/fpsyg.2025.1697168)
Supplement: Supplementary file 2 [file Table_2.docx]

**Madde ve Davranışsal Bağımlılık Tarama Testi (MDBT)**

1. **"Çok fazla yaptım"** ifadesini düşünün. Son 12 ayda, bu sizin için ne sıklıkta geçerliydi:

a) Alkol kullanımı *(bira, şarap ve/veya sert içki)*

| Hiçbir zaman | Nadiren | Bazen | Çoğu zaman | Her zaman | Bunu hiç yapmadım | Bilmiyorum / Söylemek istemiyorum |
| --- | --- | --- | --- | --- | --- | --- |

b) Tütün kullanımı (sigaralar, purolar, çiğneme ve tüm diğer tütün mamulleri dahil)

| Hiçbir zaman | Nadiren | Bazen | Çoğu zaman | Her zaman | Bunu hiç yapmadım | Bilmiyorum / Söylemek istemiyorum |
| --- | --- | --- | --- | --- | --- | --- |

c) Esrar kullanımı (marihuana, haşhaş, haşhaş yağı, ot ve diğer esrar mamulleri dahil)

| Hiçbir zaman | Nadiren | Bazen | Çoğu zaman | Her zaman | Bunu hiç yapmadım | Bilmiyorum / Söylemek istemiyorum |
| --- | --- | --- | --- | --- | --- | --- |

d) Kokain kullanımı (taş kokain, toz kokain, burundan çekmek dahil)

| Hiçbir zaman | Nadiren | Bazen | Çoğu zaman | Her zaman | Bunu hiç yapmadım | Bilmiyorum / Söylemek istemiyorum |
| --- | --- | --- | --- | --- | --- | --- |

e) Kumar *(kumar makineleri, çevrimiçi kumar, kumarhane oyunları, piyangolar, kazı kazan biletler ve para karşılığı oynanan diğer bahisler dahil)*

| Hiçbir zaman | Nadiren | Bazen | Çoğu zaman | Her zaman | Bunu hiç yapmadım | Bilmiyorum / Söylemek istemiyorum |
| --- | --- | --- | --- | --- | --- | --- |

f) Alışveriş *(mağazada ve online alışveriş dahil)*

| Hiçbir zaman | Nadiren | Bazen | Çoğu zaman | Her zaman | Bunu hiç yapmadım | Bilmiyorum / Söylemek istemiyorum |
| --- | --- | --- | --- | --- | --- | --- |

g) Video oyun *(X-Box, Wii, Playstation gibi video oyunları ve diğer çevrim içi veya çevrim dışı video oyunları dahil)*

| Hiçbir zaman | Nadiren | Bazen | Çoğu zaman | Her zaman | Bunu hiç yapmadım | Bilmiyorum / Söylemek istemiyorum |
| --- | --- | --- | --- | --- | --- | --- |

h) Fazla yemek *(günlük yaşam için gerekenden daha fazla)*

| Hiçbir zaman | Nadiren | Bazen | Çoğu zaman | Her zaman | Bunu hiç yapmadım | Bilmiyorum / Söylemek istemiyorum |
| --- | --- | --- | --- | --- | --- | --- |

i) Cinsel etkinlik *(çevrimiçi veya çevrimdışı, aşırı cinsel aktivite ve/veya uygunsuz pornografi kullanımı)*

| Hiçbir zaman | Nadiren | Bazen | Çoğu zaman | Her zaman | Bunu hiç yapmadım | Bilmiyorum / Söylemek istemiyorum |
| --- | --- | --- | --- | --- | --- | --- |

j) Fazla çalışma *(ücretli veya gönüllü)*

| Hiçbir zaman | Nadiren | Bazen | Çoğu zaman | Her zaman | Bunu hiç yapmadım | Bilmiyorum / Söylemek istemiyorum |
| --- | --- | --- | --- | --- | --- | --- |

2. **“Bir kere başladım, duramadım”** ifadesini düşünün. Son 12 ayda, bu sizin için ne sıklıkta geçerliydi:

a) Alkol kullanımı (bira, şarap ve/veya sert içki)

| Hiçbir zaman | Nadiren | Bazen | Çoğu zaman | Her zaman | Bunu hiç yapmadım | Bilmiyorum / Söylemek istemiyorum |
| --- | --- | --- | --- | --- | --- | --- |

b) Tütün kullanımı (sigaralar, purolar, çiğneme ve tüm diğer tütün mamulleri dahil)

| Hiçbir zaman | Nadiren | Bazen | Çoğu zaman | Her zaman | Bunu hiç yapmadım | Bilmiyorum / Söylemek istemiyorum |
| --- | --- | --- | --- | --- | --- | --- |

c) Esrar kullanımı (marihuana, haşhaş, haşhaş yağı, ot ve diğer esrar mamulleri dahil)

| Hiçbir zaman | Nadiren | Bazen | Çoğu zaman | Her zaman | Bunu hiç yapmadım | Bilmiyorum / Söylemek istemiyorum |
| --- | --- | --- | --- | --- | --- | --- |

d) Kokain kullanımı (taş kokain, toz kokain, burundan çekmek dahil)

| Hiçbir zaman | Nadiren | Bazen | Çoğu zaman | Her zaman | Bunu hiç yapmadım | Bilmiyorum / Söylemek istemiyorum |
| --- | --- | --- | --- | --- | --- | --- |

e) Kumar *(kumar makineleri, çevrimiçi kumar, kumarhane oyunları, piyangolar, kazı kazan biletler ve para karşılığı oynanan diğer bahisler dahil)*

| Hiçbir zaman | Nadiren | Bazen | Çoğu zaman | Her zaman | Bunu hiç yapmadım | Bilmiyorum / Söylemek istemiyorum |
| --- | --- | --- | --- | --- | --- | --- |

f) Alışveriş *(mağazada ve online alışveriş dahil)*

| Hiçbir zaman | Nadiren | Bazen | Çoğu zaman | Her zaman | Bunu hiç yapmadım | Bilmiyorum / Söylemek istemiyorum |
| --- | --- | --- | --- | --- | --- | --- |

g) Video oyun *(X-Box, Wii, Playstation gibi video oyunları ve diğer çevrim içi veya çevrim dışı video oyunları dahil)*

| Hiçbir zaman | Nadiren | Bazen | Çoğu zaman | Her zaman | Bunu hiç yapmadım | Bilmiyorum / Söylemek istemiyorum |
| --- | --- | --- | --- | --- | --- | --- |

h) Fazla yemek *(günlük yaşam için gerekenden daha fazla)*

| Hiçbir zaman | Nadiren | Bazen | Çoğu zaman | Her zaman | Bunu hiç yapmadım | Bilmiyorum / Söylemek istemiyorum |
| --- | --- | --- | --- | --- | --- | --- |

i) Cinsel etkinlik *(çevrimiçi veya çevrimdışı, aşırı cinsel aktivite ve/veya uygunsuz pornografi kullanımı)*

| Hiçbir zaman | Nadiren | Bazen | Çoğu zaman | Her zaman | Bunu hiç yapmadım | Bilmiyorum / Söylemek istemiyorum |
| --- | --- | --- | --- | --- | --- | --- |

j) Fazla çalışma *(ücretli veya gönüllü)*

| Hiçbir zaman | Nadiren | Bazen | Çoğu zaman | Her zaman | Bunu hiç yapmadım | Bilmiyorum / Söylemek istemiyorum |
| --- | --- | --- | --- | --- | --- | --- |

3. **“Bir şeyler yapabilmek için onu yapmam gerektiğini hissettim”** ifadesini düşünün Son 12 ayda, bu sizin için ne sıklıkta geçerliydi:

a) Alkol kullanımı (bira, şarap ve/veya sert içki)

| Hiçbir zaman | Nadiren | Bazen | Çoğu zaman | Her zaman | Bunu hiç yapmadım | Bilmiyorum / Söylemek istemiyorum |
| --- | --- | --- | --- | --- | --- | --- |

b) Tütün kullanımı (sigaralar, purolar, çiğneme ve tüm diğer tütün mamulleri dahil)

| Hiçbir zaman | Nadiren | Bazen | Çoğu zaman | Her zaman | Bunu hiç yapmadım | Bilmiyorum / Söylemek istemiyorum |
| --- | --- | --- | --- | --- | --- | --- |

c) Esrar kullanımı (marihuana, haşhaş, haşhaş yağı, ot ve diğer esrar mamulleri dahil)

| Hiçbir zaman | Nadiren | Bazen | Çoğu zaman | Her zaman | Bunu hiç yapmadım | Bilmiyorum / Söylemek istemiyorum |
| --- | --- | --- | --- | --- | --- | --- |

d) Kokain kullanımı (taş kokain, toz kokain, burundan çekmek dahil)

| Hiçbir zaman | Nadiren | Bazen | Çoğu zaman | Her zaman | Bunu hiç yapmadım | Bilmiyorum / Söylemek istemiyorum |
| --- | --- | --- | --- | --- | --- | --- |

e) Kumar *(kumar makineleri, çevrimiçi kumar, kumarhane oyunları, piyangolar, kazı kazan biletler ve para karşılığı oynanan diğer bahisler dahil)*

| Hiçbir zaman | Nadiren | Bazen | Çoğu zaman | Her zaman | Bunu hiç yapmadım | Bilmiyorum / Söylemek istemiyorum |
| --- | --- | --- | --- | --- | --- | --- |

f) Alışveriş *(mağazada ve online alışveriş dahil)*

| Hiçbir zaman | Nadiren | Bazen | Çoğu zaman | Her zaman | Bunu hiç yapmadım | Bilmiyorum / Söylemek istemiyorum |
| --- | --- | --- | --- | --- | --- | --- |

g) Video oyun *(X-Box, Wii, Playstation gibi video oyunları ve diğer çevrim içi veya çevrim dışı video oyunları dahil)*

| Hiçbir zaman | Nadiren | Bazen | Çoğu zaman | Her zaman | Bunu hiç yapmadım | Bilmiyorum / Söylemek istemiyorum |
| --- | --- | --- | --- | --- | --- | --- |

h) Fazla yemek *(günlük yaşam için gerekenden daha fazla)*

| Hiçbir zaman | Nadiren | Bazen | Çoğu zaman | Her zaman | Bunu hiç yapmadım | Bilmiyorum / Söylemek istemiyorum |
| --- | --- | --- | --- | --- | --- | --- |

i) Cinsel etkinlik *(çevrimiçi veya çevrimdışı, aşırı cinsel aktivite ve/veya uygunsuz pornografi kullanımı)*

| Hiçbir zaman | Nadiren | Bazen | Çoğu zaman | Her zaman | Bunu hiç yapmadım | Bilmiyorum / Söylemek istemiyorum |
| --- | --- | --- | --- | --- | --- | --- |

j) Fazla çalışma *(ücretli veya gönüllü)*

| Hiçbir zaman | Nadiren | Bazen | Çoğu zaman | Her zaman | Bunu hiç yapmadım | Bilmiyorum / Söylemek istemiyorum |
| --- | --- | --- | --- | --- | --- | --- |

4. **“Soruna yol açsa da onu yapmaya devam ettim”** ifadesini düşünün. Son 12 ayda, bu sizin için ne sıklıkta geçerliydi:

a) Alkol kullanımı (bira, şarap ve/veya sert içki)

| Hiçbir zaman | Nadiren | Bazen | Çoğu zaman | Her zaman | Bunu hiç yapmadım | Bilmiyorum / Söylemek istemiyorum |
| --- | --- | --- | --- | --- | --- | --- |

b) Tütün kullanımı (sigaralar, purolar, çiğneme ve tüm diğer tütün mamulleri dahil)

| Hiçbir zaman | Nadiren | Bazen | Çoğu zaman | Her zaman | Bunu hiç yapmadım | Bilmiyorum / Söylemek istemiyorum |
| --- | --- | --- | --- | --- | --- | --- |

c) Esrar kullanımı (marihuana, haşhaş, haşhaş yağı, ot ve diğer esrar mamulleri dahil)

| Hiçbir zaman | Nadiren | Bazen | Çoğu zaman | Her zaman | Bunu hiç yapmadım | Bilmiyorum / Söylemek istemiyorum |
| --- | --- | --- | --- | --- | --- | --- |

d) Kokain kullanımı (taş kokain, toz kokain, burundan çekmek dahil)

| Hiçbir zaman | Nadiren | Bazen | Çoğu zaman | Her zaman | Bunu hiç yapmadım | Bilmiyorum / Söylemek istemiyorum |
| --- | --- | --- | --- | --- | --- | --- |

e) Kumar *(kumar makineleri, çevrimiçi kumar, kumarhane oyunları, piyangolar, kazı kazan biletler ve para karşılığı oynanan diğer bahisler dahil)*

| Hiçbir zaman | Nadiren | Bazen | Çoğu zaman | Her zaman | Bunu hiç yapmadım | Bilmiyorum / Söylemek istemiyorum |
| --- | --- | --- | --- | --- | --- | --- |

f) Alışveriş *(mağazada ve online alışveriş dahil)*

| Hiçbir zaman | Nadiren | Bazen | Çoğu zaman | Her zaman | Bunu hiç yapmadım | Bilmiyorum / Söylemek istemiyorum |
| --- | --- | --- | --- | --- | --- | --- |

g) Video oyun *(X-Box, Wii, Playstation gibi video oyunları ve diğer çevrim içi veya çevrim dışı video oyunları dahil)*

| Hiçbir zaman | Nadiren | Bazen | Çoğu zaman | Her zaman | Bunu hiç yapmadım | Bilmiyorum / Söylemek istemiyorum |
| --- | --- | --- | --- | --- | --- | --- |

h) Fazla yemek *(günlük yaşam için gerekenden daha fazla)*

| Hiçbir zaman | Nadiren | Bazen | Çoğu zaman | Her zaman | Bunu hiç yapmadım | Bilmiyorum / Söylemek istemiyorum |
| --- | --- | --- | --- | --- | --- | --- |

i) Cinsel etkinlik *(çevrimiçi veya çevrimdışı, aşırı cinsel aktivite ve/veya uygunsuz pornografi kullanımı)*

| Hiçbir zaman | Nadiren | Bazen | Çoğu zaman | Her zaman | Bunu hiç yapmadım | Bilmiyorum / Söylemek istemiyorum |
| --- | --- | --- | --- | --- | --- | --- |

j) Fazla çalışma *(ücretli veya gönüllü)*

| Hiçbir zaman | Nadiren | Bazen | Çoğu zaman | Her zaman | Bunu hiç yapmadım | Bilmiyorum / Söylemek istemiyorum |
| --- | --- | --- | --- | --- | --- | --- |

Not: MDBT Türkçe versiyonunun kullanımı için ayrıca yazarlardan izin alınmasına gerek yoktur.
